# AI and Patient Trust in Health Care

**DOI:** 10.2196/104760

**Published:** 2026-08-21

**Authors:** Sara L Jackson

**Affiliations:** 1 Department of Medicine, Division of General Internal Medicine, University of Washington, 325 9th Ave Harborview Medical Center, Seattle, WA, 98104, United States

**Keywords:** artificial intelligence, patient concerns, physician-patient relationship, data privacy, medical ethics, health care equity

## Abstract

Health care providers are among the most trusted professionals, and they are rapidly adapting to AI integration in medicine. Authors Hou et al reviewed and synthesized qualitative studies of patient concerns regarding AI in health care. Themes included privacy, data security, and the “black box” complexity of AI decision-making; decreased trust in the physician-patient relationship and in the accountability of health systems; and equitable access, ethical regulation, and the displacement of human workers. Global and national health leaders can support health systems by establishing specific guidelines for informed consent about AI use in patient care. Similarly, health care leadership groups, in collaboration with AI developers, should establish checkpoints for physician review and specific loci for accountability prior to clinical use. Equitable access, ethical regulation, and the preservation of access to human providers, particularly when empathetic holistic care is paramount, will all impact the future of patient trust in physicians and health care systems. Centering our actions on the concerns of patients provides a road map to improve health care delivery, with AI at the service of patients and physicians.

We are trusted, we are confided in, we have become very much as secular confessors and can listen, if we choose to take the time, to the nuts and bolts that make each one of us unique....

[Sir William Osler]

This is a commentary on “Patient Concerns Regarding Artificial Intelligence Applications in Health Care: Systematic Review and Meta-Synthesis Based on Social Ecological Theory” by Hou et al [1].

Trust is the central currency at stake in AI implementation decisions in health care. In 2010, a technologic, patient-centered innovation study (open notes) allowed patients digital access to their primary care visit notes and found that patients overwhelmingly appreciated transparent access to their health information, which increased trust in the physician-patient relationship [2]. Researchers are now studying large language models to see if they improve patients’ engagement in and understanding of their health information [3]. This evolution of digital tools has the potential to continue improving the patient experience and quality of care. As we take this next step in AI transformation, listening to patients and their concerns is our North Star for maintaining trust in physicians and the health care system.

Using social ecological theory, Hou et al [1] analyzed 25 qualitative studies about patient concerns regarding AI in health care, across diverse patient groups and multiple countries. At the individual level—or microlevel—privacy and data security, as well as the “black box” complexity of decision-making, were primary themes. Mesolevel themes included patient concerns about the future degradation of physician-patient relationships and the loss of trust and accountability in health organizations. On a macrolevel—or societal level—patients voiced concerns about equitable access to new AI technology, the ethical regulation of AI tools, and the diffusion of AI such that human workers are replaced. This commentary discusses recommendations for addressing patient concerns, acknowledges the complexity and necessity, and recognizes that in medicine—more so than in other industries—patient trust is critical for therapeutic relationships and engagement.

Hou et al [1] noted “…the goal of policy intervention should not be to ‘correct patient misconceptions,’ but rather to substantively address these well-founded and evidence-based concerns,” including privacy, data security, and opacity in complex decision-making. In 2025, there were 772 health care data breaches—the largest annual volume to date [4]. Legal actions related to AI note-writing software [5] and insurers using AI algorithms to deny medical claims [6] are concrete expressions of patient concerns. Consent and transparency are key considerations when implementing AI tools in clinical settings. Establishing mandatory “physician checkpoints” within AI workflows, such that physicians review and communicate important clinical decisions, and clear loci of responsibility between providers, health systems, and AI vendors will improve transparency and create standards that patients can trust. Hou et al [1] recommended having patients consent to AI use, ideally before the clinical visit, and allowing patients to request a human review of AI decisions. These actions will improve AI transparency. In practice however, up-front consent can be a challenge, given the varied types and rapid adoption of AI products. Patients could opt out of some applications, such as AI note-writing scribes, but other AI tools may be incorporated into back-office or data analytic functions that cannot be turned off for individual patients. The complexity of accurately communicating true informed consent to patients across a spectrum of digital and health literacy is another barrier. Global and national collaborative efforts to support health systems in developing standardized informed consent would support clarity related to AI use in health care.

The World Health Organization’s (WHO’s) 2025 guidance on the ethics and governance of AI for health highlights core principles, including protecting autonomy, promoting human well-being, ensuring transparency, fostering accountability, ensuring equity, and promoting AI that is responsive and sustainable [7]. Hou et al [1] discussed that equitable access to beneficial AI tools will require regulation to encourage investment in low-income populations and remote areas, as well as the provision of tools that are easy to use and low-cost. How these aspirational equity goals will be affected by cost and the data centers and energy required to support AI has yet to be determined. The ethical implications of AI energy consumption and the impact upon climate are key areas for transparency and regulation, given the known human health consequences from severe climate events. Cost is another factor that undermines trust in physicians and health systems [8]. AI vendors have clear financial incentives for the use of their products. Human labor is expensive for health systems and can potentially be reduced by the implementation of AI. Unintended consequences of the rapid adoption of AI in health care, within the areas of equity, energy and climate, cost, and human resources, are of concern to patients.

Trust in US physicians and hospitals dropped from 72% in 2020 to 40% in 2024, after the COVID-19 pandemic [9]. This was, in part, related to the internet amplifying conflicting medical advice. AI has the potential to promote information that is not evidence based and further erode patient confidence in physicians. Patients often wait weeks or months to see physicians. AI could efficiently fill a clinical gap for specific, predetermined scenarios if it proves accurate and acceptable. However, trust is fostered by human empathy and longitudinal relationships. It will be harder for patients to trust doctors and health systems if they have limited access to human providers. In longitudinal relationships, such as those in primary care, or in highly emotional situations, such as a new complex diagnosis or the end of life, access to human empathy should be the default.

In health care, our mission is to better the lives of humans. We should be held to a higher standard than other sectors when it comes to vetting the use of AI. Do we rapidly adopt disruptive innovation, hoping for dramatic improvements in experience, quality, and costs, or do we take time to assess benefits vs risks, particularly in the areas that concern patients? Osler’s advice reminds us that taking the time to listen to our patients will go a long way toward earning their trust as we navigate the AI landscape in medicine.

## Abbreviations

WHO: World Health Organization
